# Assessing perforation of acute appendicitis using the delta neutrophil index reflecting the peripheral immature granulocyte count

**DOI:** 10.1186/cc10671

**Published:** 2012-03-20

**Authors:** NG Rhee, S Chung

**Affiliations:** 1 Yonsei University College of Medicine, Seoul, South Korea

## Introduction

The delta neutrophil index corresponds to the calculated immature granulocyte counts and the severity of sepsis. This study investigated the diagnostic value of the delta neutrophil index as a preoperative laboratory marker for appendiceal perforation in patients with acute appendicitis.

## Methods

This study was a retrospective analysis of patients confirmed as appendicitis pathologically from November 2009 to September 2010 at two hospitals. The delta neutrophil index was automatically calculated as a subset of routine complete blood count test. The diagnostic performance of the delta neutrophil index for perforated appendicitis was evaluated.

## Results

During the study period, 308 patients were enrolled. Among them, 32 patients (10.4%) were confirmed as perforated appendicitis. The delta neutrophil index was significantly higher in the perforated group than the nonperforated group (4.8 ± 7.1% vs. 2.0 ± 2.0%, *P *< 0.05). The sensitivity and specificity of the delta neutrophil index for predicting perforated appendicitis was 25.0% and 96.7% respectively at a cutoff level of 5% with an area under the curve of 0.78 on the ROC curve.

## Conclusion

This study suggested that the delta neutrophil index is associated with perforated appendicitis. However, the sensitivity was not high enough to use as a clinical guidance.
